# Prevalence of problematic cell phone use in an adult population in Spain as assessed by the Mobile Phone Problem Use Scale (MPPUS)

**DOI:** 10.1371/journal.pone.0181184

**Published:** 2017-08-03

**Authors:** José de-Sola, Hernán Talledo, Fernando Rodríguez de Fonseca, Gabriel Rubio

**Affiliations:** 1 Department of Psychobiology, School of Psychology, Complutense University of Madrid, Somosaguas Campus, Madrid, Spain; 2 San Ignacio de Loyola University of Lima, Lima, Perú; 3 Clinical Mental Health Management Unit, Regional University Hospital of Málaga, Biomedical Research Institute of Málaga – IBIMA, Málaga, Spain; 4 Department of Psychiatry, Complutense University, Psychiatric Service (University Hospital 12 of October), Madrid, Research Institute i+12, Addictive Disorder Network (RETIS), Madrid, Spain; Ariel University, ISRAEL

## Abstract

Problematic cell phone use has alarmingly increased in industrialized countries in the past 10 years. For many perpetrators, it can turn into a behavioural addiction, although this is not a recognized medical condition. Although there are many tools for evaluating this use, one of the most widely used tools is the Mobile Phone Problematic Use Scale (MPPUS), which we test on a representative sample of the population in Spain to obtain an estimate of the prevalence of problematic cell phone use in our midst. The age range consists of 16–65 years, with 1,126 surveys conducted. In this population, we verify that the reliability and internal consistency of the MPPUS (α = 0.939) are maintained. Additionally, the construct validity, considering the derived factors (Abuse and Dependence, Craving and Loss of Control, and Dependence on the Social Environment) are aligned with other research and with diverse external criteria of addiction. We establish four categories of users (Casual, Regular, At Risk, and Problematic) and obtain a prevalence of 15.4% among At Risk Users and 5.1% among Problematic Users. This finding implies a total of 20.5% of Users with Problems. A binary logistic regression analysis shows that age, gender, level of education, and daily cell phone use predict problematic cell phone use. The results, based on multiple criteria, show that such problematic use shares features of recognized addictions, affecting large segments of the population and not only adolescents.

## Introduction

The need for social contact and relationships with other people is an essential part of human nature. Communication styles have evolved throughout history to the most modern means such as cell phones. Even these have steered towards the most sophisticated applications, in which, presently, texts are more important than actual calls. Furthermore, there has been a shift from interpersonal communication to simultaneous group communication mostly in written form, in which the written word substitutes for oral conversations. A recent study shows that, currently, there already are more cell phones than people. In 2016, the percentage of cell phones categorized as smartphones in Europe was 78% whereas, in Spain, it was 87%. We already have more smartphones than computers. On average, a Spaniard uses his or her smartphone for approximately three and a half hours every day, with email, instant messaging, and surfing the web being the main uses. In turn, the age of first use is constantly decreasing, with 98% of adolescents (10–14 years of age) owning their next generation data terminal [1], even though at two and three years of age, most play with their parents’ cell phones [2,3].

Indeed, having acell phone is already a way of being in the world. Many users remarks that “without having a cell phone, one feels like if one does not exist”. Additionally, this reality has been moulded it into an excellent channel of expression, a distinguishing mark in which problematic usage would be a projection of personal traits and styles of group interactions. Although from a marketing and advertising perspective, the cell phone revolution would be a manifestation of a new social tendency and a new market opportunity, for the clinical world, it presents a problem that affects millions of people, in which, ironically, a tool designed to improve social contact ultimately interferes with social contact [4]. However, problematic cell phone use is not a recognized medical condition and has no delineated symptom profile, In addition, there is a recognized influence of sociocultural factors determining the type of problematic cell phone use, and a multiplicity of approaches for its evaluation [for review see references 3–5].

From this perspective, currently, the use of cell phones is no longer necessarily associated with our voice but is focused on applications such as social networks or chatting, e.g., WhatsApp. In other words, new user profiles have been created that are detached from spoken communication and consistent with diverse, essentially interactive, applications, and we are now aware of the existence of abuse, with the subsequent abandonment or inappropriate use and interference with daily activities and healthy habits [5].

The extent and usage of these devices are so pronounced that some investigators have come to consider the cell phone to be one of the greatest addictions of the current century [6]. In this sense, physical disorders [7], usage in dangerous situations [8], social and family problems with a loss of interest in other activities and personal contact [9–16], sleep disorders [17,18], urgency and difficulty of control [19–21], and anxiety and irritability have been suggested when the data terminal is unavailable [22–24]. However it is important to insist in the novelty of the problem and the lack of medical recognition—i.e. international agreement on the the health problems derived of problematic cell phone use—which demands more research to define the real consequences of excessive cell phone use.

In general, technology has a powerful potential for diversion and escape, which can potentially make it addictive. Furthermore, reliance on communication and social contact is added thereto, particularly among young people. In fact, the influence of friends or the environment, social relationships, and the need for social belonging are factors that change a cell phone into a potent device that is capable of satisfying these needs. This phenomenon would also explain the urgency, dependency, and feeling of loneliness when the terminal is unavailable, the compulsive use of applications [25,26], or the need to overcome boredom by looking for new sensations [20,27]. For their part, Foersteret al.[28]suggested two behavior patterns in the problematic use of cell phones: entertainment in surfing the web and social contact. Regarding the former, the smartphone has replaced the PC as the access element to the Internet, and it currently provides a plethora of applications with a great potential for entertainment. Regarding the latter, there is the need for connection, for interconnectivity, the need to not feel left out that would satisfied be by the cell phone.

Historically, the first studies were launched in 2004, with the publication of the Cellular Phone Dependence Questionnaire (CPDQ) [29]. In Spain, Muñoz-Rivas and Agustín[30] raised alarm bells in due course, referring to “cell phone addicts” and deeming young people to be at highest risk. Since then, given the significant number of published evaluation tools, the data on prevalence vary and are scattered, most likely due to the methodological diversity, samples, and criteria of those that have been designed.

Nonetheless, the currently available reference scale, the Mobile Phone Problem Use Scale (MPPUS) by Bianchi and Phillips [31] emerged in 2005 and is used in the present study. Part of the criteria for technological addictions [32], were designed to easily evaluate a pattern of problematic usage, though some authors use the terms addiction and dependence interchangeably in this tool [33].

Despite the time that has passed, the MPPUS continues to be one of the reference tools, validated and supported by a multitude of research in different countries. Although it was designed for an adult population, it has been adapted to specific groups, as in the case of the Mobile Phone Addiction Index (MPAI) for American adolescents and youth aged 14 to 28 years [20,27], the MPPUSA (Mobile Phone Problem Use Scale) for Spanish and British adolescents [34,35], and the MPPUS-10 intended for Swiss adolescents aged 12 to 17 years, with a reduction in the original scale to 10 items [28], in addition to university populations in Teheran [36], in Japan [37], or in Germany, aged 18 to 46 years, in which the original Likert scale is adjusted from 10 to five points [33].

The objective of this study is to validate the MPPUS in a representative population in Spain, including adolescents and adults, and to determine the prevalence of problematic users in the general population. Simultaneously, considering various sociodemographic, cell phone use and drug use variables, we analyse which variables have predictive value for problematic use and which variables can simply be associated with it.

The intention of the present study is to have a view of the dimension of problematic cell phone use in Spain, its presence beyond the adolescence and the influence of gender, education, patterns of use, social networks etc. Although youth is a time period in which cell phones can cause important problems, these same problems would also exist in adulthood [38] ans this information, which we lack, is relevant for further analysis of the problem. Finally, this research will be very valuable for further comparisons of problematic cell phone use with recognized drug or behavioral addictions, helping to establish its potential medical condition eventually determining health interventions.

## Materials and methods

### Procedures

With e-mail invitations sent between January and December 2014, an on-line survey with a semi-structured questionnaire was conducted. This questionnaire was piloted with 10 participants in paper format, with their results having been excluded from the final sample. Each participant was given a link that allowed him or her to access a website from which the survey was launched. The online survey program Internet SSI Web edition 6.8 by Sawtooth Software was used. The interviewee could suspend and return to the survey at his or her leisure. The link was inactivated once the survey was complete. Each participant had to have his or her own cell phone. A point that was evaluated by an initial filter question predetermined the continuation of the survey.

Approximately 20% of the sample was obtained via submissions performed by the research team. The rest was obtained by a company specializing in on-line surveys and sociological research, which used its database of 151,170 individuals in Spain. Finally, a sample of 1,126 valid surveys out of a total of 1,600 submissions was obtained. In both recruitment arms, the survey was exactly the same.

The survey comprises elements of broader research; the following are used in this study:

1. Sociodemographic variables, aspects and usage of cell phone, and drug use. Age, gender, city and autonomous community in Spain, level of education of the participant and of his or her parents, main employment, and drug use (legal or illegal) are considered. Simultaneously, assessments of the quality of the device, the time owning a cell phone, daily usage, and the number of friends with whom one maintains contact via cell phone (Appendix I) are included.
2. The Mobile Phone Problematic Use Scale (MPPUS). This instrument was originally designed and validated by Bianchi and Phillips [31] for a sample of aged 18 to 85 years; it has 27 Likert-type items and response options ranging from 1 (not true at all) to 10 (totally true). There are versions in which the number of items has been adapted or the response range has been reduced to 1 to 5 [33,36,39]. The total score for the original scale considers a range of 27 to 270 points. However, in our case, we use the adaptation by López-Fernández et al. [34] for the general adolescent population in Spain, in which the original 27 items are reduced to 26 by eliminating item 4 (Appendix II).

### Ethics statement

The present project was approved by the Ethics Committee of i+12 Institute in Madrid. The study is a web platform-based survey with includes an inform consent at the beginning of the questionnaire where the participants are informed of the purpose of the study. All subjects gave their free acceptance in accordance with the Declaration of Helsinki. All the procedures guarantee the generation of completely anonymized datasets.

### Statistical analysis

We employed SPSS, versions 23 and 24 (IBM, Armonk, NY, USA), for the statistical analysis.

An internal consistency analysis of the MPPUS was conducted by means of Cronbach’s alpha coefficient [40]. An exploratory factor analysis (EFA) was performed to verify and validate the internal structure in relation to others. Similarly, we established cut-off points for four categories of users (Casual, Regular, At Risk, and Problematic) to determine their prevalence in relation to the sociodemographic variables used. These four categories were further classified into Normal Users (Casual and Regular) and Problematic Users (At Risk and Problematic) in their use as dependent variables, with objective of identifying the questionnaire items that may have a more significant contribution to the final score on the at risk + problematic use population, as we discuss below.

An analysis of frequencies and averages is presented, with intersections for sociodemographic data, drug use, and Problematic Users of the MPPUS. Significant differences were calculated using analysis of variance (ANOVA) for average scores and the chi-squared test for frequencies. Similarly, correlation analyses were performed on problematic use measured with the MPPUS. Kendall’s tau coefficient was used with ordinal variables and Pearson’s coefficient with continuous variables.

The closed-ended questions in the survey, with a score ranging from 1 to 5, were recoded and simplified in three response categories. Furthermore, age was categorized into 10-year intervals to facilitate the frequency analysis but nonetheless using it as a continuous variable in the correlation analysis.

Finally, we performed a binary logistic regression analysis to determine the influence of the sociodemographic variables (age, gender, the level of education of the participant and of his or her parents), drug use, daily usage, cell phone quality, the time period the participant has owned a cell phone, and the number of friends with whom the user maintains contact via cell phone) on the two categories (Regular Users and Problematic Users). This step was based on a review of the previous research.

In all cases, the level of maximum permissible significance was at a confidence level of 5%.

### Sample and participants

The sample consisted of 1,126 Spanish survey respondents from the entire country, men and women between 16 and 65 years of age. The sampling was conducted via a non-probabilistic quota selection with geographical proportionality to the population size of each of the 17 autonomous communities in Spain (except Ceuta and Melilla) and according to 2014 data from the National Institute for Statistics. Slightly more than half of the surveys were conducted in provincial capitals and main centres with 100,000 inhabitants (54.8%), whereas the remainder corresponded to rural areas and small urban areas (45.2%).

Regarding age, equal segments of ages 16 to 25 years and 26 to 35 years were established with the intent to obtain information that can be compared to that from other studies. The average age of the sample population was 32.8 years, with a standard deviation of 11.67. Of those, 47.7% were men and 53.3% women.

Regarding main occupation, it was found that slightly more than half of the participants were employed (57.3%), with the remainder being either unemployed (20.2%), students (18.7%), or homemakers (3.8%). The level of higher education (university graduates or bachelor degree) had a significant weight compared to the rest (63.5%); more than one-fourth had attained secondary education (30.4%), whereas a minority had not attained elementary or basic studies (6.1%). Comparatively, the level of parental education was centred on basic studies (40.4%), followed by higher education (28.4%) and secondary education (27.0%), with a small percentage of parents without education (4.2%).

Finally, general drug of abuse use was at 50.7%, with 5.5% being illegal drug use compared to 45.2% being legal drug use, with an average total time of use of 10 years (M = 10.3 years, SD = 6.73). See Table 1 for general information.

**Table 1:** Distribution of the sample with respect to geographic area, age, gender, main occupation, and level of schooling.

**Autonomous Communities**		**Age**		**Schooling**	
Andalusia	15.7%	16 to 25 years	40.9%	Higher Education	63.5%
Aragon	2.5%	26 to 35 years	24.0%	Middle Education	30.4%
Asturias	2.0%	36 to 45 years	17.0%	Basic Education	6.1%
Balearic Islands	1.9%	46 to 55 years	13.1%	No Schooling	4.2%
Canary Islands	3.9%	56 to 65 years	5.0%		
Cantabria	1.2%			**Drug of abuse use (use ever)**	
Castilla La Mancha	3.9%	**Gender**		Alcohol	43.5%
Castilla y Leon	4.4%	Male	47.7%	Tobacco	19.6%
Catalonia	13.1%	Female	53.3%	Cannabis and/or Psychostimulants	5.5%
Extremadura	2.3%				
Galicia	5.0%				
La Rioja	0.8%	**Main Occupation**			
Madrid	26.2%	Worker	57.3%		
Murcia	2.5%	Unemployed	20.2%		
Navarra	1.1%	Student	18.7%		
Basque Country	3.5%	Household duties	3.8%		
Valencia	10.0%				

Frequencies obtained from the total sample (1,126 interviews)

## Results

### Internal validity of the MPPUS: Exploratory factor analysis

When considering the 26 items of the MPPUS that were ultimately used, an EFA was performed with the original range of 1 to 10 points. The minimal score was 26, and the maximum score 260, with a higher score indicating a more problematic use. The average was 68.9, with a standard deviation of 36.89, a median of 59, and a range of 234. The extraction method was performed with a principal component analysis with Varimax rotation and Kaiser normalization.

The EFA provided us with four factors or components that explain 59.8% of the total variance, with intrinsic value above 1 (Table 2). The final rotation converged in the interations described in the table. All items maintained factorial loads above 0.4, with the measure being the Kaiser-Meyer-Olkin sample adequacy of 0.96 (KMO = 0.96), whereas Bartlett’s test of sphericity provided a chi-square of 16666.964 (df = 325; p<0.000). This finding confirms the sample adequacy and the suitability of the analysis.

**Table 2:** Exploratory factor analysis of the 26 MPPUS items in a Spanish adult and adolescent population.

	Matrix of rotated factors			
	1	2	3	4
P.14 I tend to dream of the phone.	.811			
P.22 I get in a bad mood if I have to turn off my phone during class, at meal times, or at the movies.	.803			
P.21 I tend to be late for appointments because I am hooked on my phone when I shouldn’t be.	.800			
P.4 I have tried to hide from others the amount of time I spend talking on the phone.	.695			
P.17 My performance has suffered due to the amount of time I spend on the phone.	.671			
P.18 I have issues that are associated with my cell phone use.	.670			
P.23 I have been told that I spend too much time on the phone.	.658			
P.15 My friends and family complain because I use the phone too much.	.646			
P.20 Sometimes, I prefer to use my phone rather than deal with other more urgent matters.	.645			
P.24 I have been in trouble more than once because my phone rang during class, at the movies, or at the theatre	.609			
P.6 I have spent more time than I should or could afford with the phone.	.588			
P.3 I spend time on my phone when I should be doing other things, which causes problems.		.714		
P.5 Cell phone use has taken away hours of my sleep.		.668		
P.2 When I feel bad, I use my phone to make myself feel better.		.658		
P.19 I am hooked on my phone for more time than I would like to be.		.621		
P.10 I have used my phone to talk to others when I felt lonely or isolated.		.600		
P.9 The time I spend on the phone has increased in the past 12 months.		.588		
P.11 I have tried to spend less time on the phone but have been unable to so.		.548		
P.8 Occasionally, while talking on the phone and engaged in an activity, I lose track of what I am doing due to the phone conversation.		.465		
P.7 I worry that I miss calls if I am not reachable			.700	
P.16 If I didn’t have a phone, my friends would have a hard time getting in touch with me.			.645	
P.26 I feel lost without my phone.			.606	
P.12 I have a hard time turning the phone off.			.543	
P.13 I feel nervous if I haven’t checked messages or if I haven’t turned on the phone.			.543	
P.25 My friends don’t like it if my phone is turned off.			.540	
P.1 I never have enough time for the phone.				.657
RESULTS	**1**	**2**	**3**	**4**
Intrinsic value of the factors or rotated components	6.738	4.570	3.197	1.043
Intrinsic value of the factors or rotated components	6.738	4.570	3.197	1.043
Percentage of explained variance of each component or factor	25.9%	17.6%	12.3%	4.0%
Accumulated percentage of explained variance for the components or factors	25.9%	43.5%	55.8%	59.8%

The data were calculated based on a sample size of 1,126 survey respondents, showing the factors, factorial loads, and explained variance of each factor.

The first factor, which comprised 25.9% of the variance with 11 items, was designated “Abuse and Excessive Phone Use”. It is defined by excessive use and recurrent thoughts (items 14 and 20), mood alterations when a data terminal is unavailable (item 22), problems or disturbances in everyday life (items 21, 17, 24, and 6), discomfort (item 18), and personal awareness of abuse or environmental warnings (items 4, 23, and 15).

The second factor, which comprised 17.6% of the variance with eight items, was designated “Loss of Control”. It involves problems derived from progressive abandonment of activities (items 3, 5, and 8), failure of control leading to use the phone more than intended (items 19, 9, and 11), or as a resource to compensate for dysphoric moods (items 2 and 10).

The third factor, which entailed 12.3% of the variance with six items, was designated “Social Context-induced Craving”. It refers to the personal perception of negative affects when the cell phone it is not available, and the need to be connected in certain relevant social settings (items 7, 16, 26, 12, 13, and 25).

The last factor actually only had one item, with a variance explained of 4% (item 1). It came to define the tendency of a progression or increase in usage of the device, that clearly can be associated with “Tolerance”.

### Internal consistency analysis

The internal reliability and consistency analysis was performed with a total Cronbach’s alpha, both for items and for averages.

In general terms, the MPPUS displays good internal consistency in our sample (α = 0.939). Based on the analysis by items, none of the cases is shown to have values below 0.935, with a correlation range of 0.940 to 0.935 (Table 3). The analysis by averages, in which correlations were calculated in two blocks [41], in our case of 13 items, provides coefficients of 0.883 and 0.904. Each half has a Guttman coefficient of 0. 893, i.e., covariance between both halves [42].

**Table 3:** Descriptive analysis and internal consistency of the items via Cronbach’s alpha coefficient.

	Average	SD	Maximum Score	Minimum Score	Cronbach’s alpha if part was omitted
P.1 I never have enough time for the phone.	2.72	2.03	10	1	.940
P.2 When I feel bad, I use the phone to make myself feel better.	3.16	2.48	10	1	.936
P.3. I spend time on the phone when I should be doing other things, which causes problems.	3.05	2.31	10	1	.936
P.4 I have tried to hide from others the amount of time I spend on the phone.	1.87	1.74	10	1	.936
P.5 Cell phone use has taken away hours of my sleep.	2.86	2.50	10	1	.936
P.6 I have spent more than I should or could afford on the phone.	1.98	1.92	10	1	.937
P.7 If I am not reachable, I worry that I miss calls.	3.45	2.65	10	1	.938
P.8 Occasionally, while talking on the phone and engaged in an activity, I lose track of what I am doing due to the phone conversation.	3.47	2.45	10	1	.937
P.9 The time I spend on the phone has increased in the past 12 months.	3.41	2.64	10	1	.937
P.10 I have used my phone to talk to others when I felt lonely or isolated.	3.91	2.82	10	1	.938
P.11 I have tried to spend less time on the phone but have been unable to do so.	2.29	2.04	10	1	.935
P.12 I have a hard time turning off the phone.	3.58	3.01	10	1	.937
P.13 I feel nervous if I haven’t checked messages or if I haven’t turned on the phone.	2.96	2.38	10	1	.935
P.14 I tend to dream of the phone.	1.53	1.48	10	1	.937
P.15 My friends and family complain because I use the phone too much.	2.22	2.05	10	1	.935
P.16 If I didn’t have a phone, my friends would have a hard time getting in touch with me.	4.53	2.93	10	1	.941
P.17 My performance has suffered due to the time I spend on the phone.	1.97	1.83	10	1	.935
P.18 I have issues that are associated with my phone use.	1.85	1.82	10	1	.936
P.19 I am attached to the phone for more time than I would like to be.	2.73	2.40	10	1	.935
P.20 Sometimes, I prefer to use the phone rather than deal with other more urgent matters.	2.04	1.87	10	1	.936
P.21 I tend to be late for appointments because I am hooked on the phone when I shouldn’t be.	1.70	1.57	10	1	.936
P.22 I get mad if I need to turn off the cell phone in class, at meal times, or at the movies.	1.70	1.66	10	1	.936
P.23 I have been told that I spend too much time on the phone.	2.22	2.16	10	1	.935
P.24 I have been in trouble more than once because my phone rang during class, at the movies, or at the theatre.	2.20	2.12	10	1	.937
P.25 My friends don’t like it if my phone is turned off.	2.78	2.49	10	1	.938
P.26 I feel lost without the phone.	2.74	2.36	10	1	.935
**SUM**	**68.9**	**36.89**	**260**	**26**	

Average values, standard deviations, and ranges by item and total sum are presented. Cronbach’s alpha is presented by item assuming that one part was omitted.

A detailed analysis per item shows a higher score in items 16 (M = 4.53, SD = 2.93) (“If I didn’t have a phone, my friends would have a hard time getting in contact with me”) and 10 (M = 3.91, SD = 2.82) (“I have used the phone to talk to others when I have felt lonely or isolated”) (Table 3).

Similarly, and considering Users with Problems (n = 231) as joined categories, i.e., the sum of the At Risk Users (n = 173) and Problematic Users (n = 58), the items with the highest scores are items 12 (M = 6.40, SD = 2.23) (“I have a hard time turning off the phone”), item 16 (M = 5.87, SD = 2.28) (“If I didn’t have a phone, my friends would have a hard time getting in contact with me”), and item 9 (M = 5.83, SD = 2.06) (“The time I spend on the phone has increased in the past 12 months”).

### Prevalence, user categories, and cut-off points in the MPPUS

We categorized users based on the following criteria by Chow et al.[43], which involve establishing four categories (Casual Users, Habitual or Regular Users, At Risk Users and Problematic Users), considering the 15, 80, and 95 percentiles as cut-off points; in our case, these categories correspond to scores of 33, 97, and 139 on the MPPUS, respectively (See Table 4).

**Table 4:** Average scores and prevalence for typical users of the MPPUS.

	Average	SD	Median	Maximum score	Minimum score	Percentage
Casual Users	28.44	2.28	28	32	26	13.6%
Regular Users	58.71	17.86	56	96	33	65.9%
At Risk Users	117.65	11.97	118	138	97	15.4%
Problematic Users	161.64	23.65	157	260	139	5.1%
**Total**	**68.9**	**36.89**	**59**	**260**	**26**	

Averages, standard deviations, medians, score ranges, and percentages of prevalence by user type and for the entire sample size of 1,126 survey respondents are presented.

Thus, the frequency of Casual Users is 13.6% (with an average score of 28.44 and a standard deviation of 2.28); Regular Users constitute 65.9% (with an average score of 58.71 and a standard deviation of 17.86); At Risk Users comprise 15.4% (with an average of 117.65 and a standard deviation of 11.97); and Problematic Users represent 5.1% (with an average of 161.64 and a standard deviation of 23.65) (Table 3). There are significant chi square differences among the four categories (chi = 1031.250^a^, df = 3, p = 0.000).

Considering Users with Problems (the sum of At Risk Users and Problematic Users) as a global category, the prevalence is 20.5%. In this case, the cut-off point is a score of 139.

#### Prevalence by age

There are significant differences in averages among the age groups, just as the ANOVA indicates (F = 29.123, df = 4, p = 0.000). The more prominent use is in the young population of 16-25-year-olds (M = 80.11, SD = 37.13) and of 26-35-year-olds (M = 69.94, SD = 38.87). Regarding the total average, the numbers invert after this age group (M = 68.95, SD = 36.89). Additionally, a negative Pearson’s correlation of use with age is also noted (r = -0.304, p = 0.000).

In terms of the percentages of prevalence, there are also significant differences among the categories of users (chi Square = 114.880, df = 12, p = 0.000). The highest prevalence is also observed in the 16-25-years age group between At Risk Users (22.6%) and Problematic Users (7.2%).

#### Prevalence by gender and level of education

Although men show a higher average score (M = 70.91, SD = 37.18) compared to women (M = 67.23, SD = 36.58) and compared to the total (M = 68.95, SD = 36.89), the differences are not significant. Neither was there a significant difference in the percentages of prevalence, though men tend to stand out as At Risk Users (16.7%) compared to women (14.2%) and compared to the total (15.4%). Similarly, there are no significant differences in the level of education of the users (neither in Higher Education, nor in Secondary nor Primary School level), for either the average scores or the percentages of prevalence.

Conversely, the parents’ level of education clearly influenced the levels of problematic cell phone use. Thus, users born from parents with lower education level have a lower mean MPPUS score (F = 4.674, df = 3, p = 0.003; i.e. Parents with higher education (M = 53.36, SD = 31.49) versus parents with no primary studies (M = 71.49, SD = 37.39)) Thus, a higher level of education of the parents is a risk factor for developing an abnormal use of the cell phone.

#### Prevalence by drug of abuse use

There are no significant differences in either the average scores or in the prevalence among users with respect to drug use. Nonetheless, among illegal drug users, the average is higher (M = 79.18, SD = 37.50) with respect to the total average (M = 68.95, SD = 36.89). Similarly, their prevalence as At Risk Users (22.6%) is also above the total for this group of users (15.4%).

### Predictive variables of problematic users

Knowing the prevalence and categories of users, we found it relevant to determine which variables are capable of predicting or discriminating between Normal Users and Problem Users by examining their sociodemographic variables, drug use, and cell phone use, which the scientific literature has highlighted in recent years.

To the end, we performed a binary logistic regression analysis including gender, age, level of education, parents’ level of education, drug use, the amount of time of phone ownership, the quality of the data terminal, the number of friends with whom the user stays in contact, and daily use as independent variables.

The dependent variables consisted of two recoded categories: Normal Users (Casual Users and Regular Users) and Problem Users (at Risk Users and Problematic Users).

The results show that only gender, level of education, daily cell phone use and age discriminate between Normal Users and Problem Users. Thus, being male, having a lower level of education, using the cell phone with a higher daily rate in hours, and being of an age of up to 35 years—and, to a lesser extent, up to 45 years—increase the probability of being a Problem User. Specifically, in our study, level of education maintains an inverse relationship with the dependent variable, in which higher education decreases the probability of problematic cell phone use compared to lower educational levels (Table 5).

**Table 5:** Binary logistic regression analysis with considered independent variables.

							95% for O.R.	
	β	E. Standard	Wald	gldf	p (sig.)	O.R.	L. Inferior	L. Superior
Number of friends	0.011	0.007	2.689	1	0.101	1.011	0.998	1.025
Level of education of parents			5.870	3	0.118			
Level of higher education of parents	0.331	0.513	0.416	1	0.519	1.392	0.509	3.805
Level of secondary education of parents	0.256	0.507	0.255	1	0.614	1.292	0.478	3.492
Level of primary education of parents	-0.123	0.498	0.061	1	0.804	0.884	0.333	2.345
Gender (males)	0.389	0.165	5.579	1	0.018*	1.475	1.068	2.037
Level of education			8.643	2	0.013*			
Higher education	-0.914	0.334	7.496	1	0.006 **	0.401	0.209	0.771
Secondary education	-0.597	0.335	3.181	1	0.075	0.550	0.286	1.061
Drug use (legal or illegal)	0.117	0.315	0.138	1	0.711	1.124	0.607	2.082
Daily cell phone usage	0.215	0.033	42.389	1	0.000 **	1.240	1.163	1.324
Quality of the data terminal	-0.158	0.290	0.298	1	0.585	0.854	0.484	1.506
Duration of cell phone ownership	-0.014	0.021	0.397	1	0.529	0.987	0.946	1.029
Age			16.291	3	0.001 **			
16–25 years	1.419	0.358	15.689	1	0.000 **	4.134	2.048	8.343
26–35 years	1.018	0.346	8.672	1	0.003 **	2.768	1.406	5.452
36–45 years	0.791	0.363	4.737	1	0.030 *	2.205	1.082	4.496
**COEFFICIENTS**								
R² Cox & Snell	Pp = 0.108							
R² of Nagelkerke	Pp = 0.169							
Hosmer &Lemeshow Cchi = 4.548 gldf = 8	Pp = 0.511							

Cox and Snell, Nagelkerke and Hosmer and Lemeshow coefficients are shown, in addition to independent variables with beta value (β), Wald, and Odds Ratios and levels of significance for at 0.05 (*) and 0.01 (**).

### Problematic cell phone use

Hence, out of all of the variables, age and daily usage time are the most important predictors. Additionally, in a different analysis, we have tested whether this last variable also maintains a positive Pearson’s correlation, which is significant for problematic cell phone use in general (r = 0.364, p = 0.000) and in all of the age subgroups (16–25 years: r = 0.321, p = 0.000; 26–35 years: r = 0.243, p = 0.000; 36–45 years: r = 0.319, p = 0.000; 46–55 years: r = 0.399, p = 0.000; 56–65 years: r = 0.344, p = 0.000). Simultaneously, and as noted above, age maintains a significant inverse relationship with problematic cell phone use.

However, the number of friends with whom one maintains contact on the phone, drug use (legal or illegal), the time that one has owned the phone, the quality of the terminal, and the parents’ level of education are not predictive variables, though the latter variable shows significant differences in prevalence, as noted above.

Nonetheless, in another analysis, we observe that the number of friends maintains a significant Pearson’s correlation between young people aged 16 to 25 years (r = 0.115, p = 0.014). This phenomenon is something that also occurs with owning a smartphone, which correlates with the scores on the MPPUS (tau = 0.112, p = 0.000). The same holds true for drug use, which, without being a predictive variable of problematic cell phone use, shows proximity to it, mainly in the group of 16-25-year-olds, in which a higher use of illegal drugs is noted (chi = 25.626, df = 8, p = 0.001).

Thus, although gender, level of education, daily cell phone use, and age predict problematic use, the remaining indicated variables maintain a relationship with this user type.

## Discussion

The objective of this study was based on the analysis of problematic cell phone use in a population aged 16 to 65 years in Spain. Our hypothesis was based on the assumption that problematic use would extend beyond the adolescent population, which is the main object of a large portion of already published research. In addition, we analyzed factors indicating whether problematic cell phone use might be comparable with other behavioral addictions.

We performed a validation of the MPPUS among a population of 16-65-year-olds in Spain, using the adaptation by López-Fernandez et al. [34] (MPPUSA) to subsequently determine the variables that can be related to it and that are capable of predicting it, once the prevalence of problematic use was known.

In general, the MPPUS has proven to be a valid tool in the detection of problematic cell phone use in a large range of ages. In our study, good internal consistency (α = 0.939) was revealed, which is in line with the results obtained by Bianchi and Phillips [31] (α = 0.93), López-Fernández et al. [34], and López-Fernández et al. [35] among Spanish and British adolescents (α = 0.97 in both cases), Takao et al.[44] and Takao [37] among university students (α = 0.89 and 0.90, respectively), Sar and Isiklar[39] among Turkish students (α = 0.94), Park and Lee [45] (α = 0.92) among Korean students aged 18 to 25 years, Kalhori et al. [36] among Iranian university students (α = 0.91), Foerster et al. [28] among Swiss adolescents (α = 0.92, and α = 0.85 in a version reduced to 10 items), and, finally, Montag et al. [33] among a population aged 18 to 46 years (α = 0.86). Although the similar internal consistency was observed in multiple wide transcultural studies using this scale, pointing to the existence of common factors in problematic cell phone use, more in depth analysis of cultural influences on the anomalous use of this technology has to be performed.

Similarly, the analysis of internal validity revealed a useful structure consisting of four factors (Excessive phone use, Abuse and Dependence, Withdrawal-craving and tolerance). This structure was found to be in agreement with that of other studies, such as the studies by Bianchi and Phillips [31], who obtained five factors involving “Tolerance”, “Flight from problems”, “Abstinence”, “Craving”, and “Adverse or negative consequences”, in a sample of 18-to-85-year-olds; Foerster et al. [28], with five factors designated “Loss of control”, “Abstinence”, “Negative consequences”, “Craving”, and “Dependence on others” among adolescents; and Kalhori et al. [36], with three factors designated “Abuse”, “Symptoms of abstinence”, and “Worry” in university students.

Users of the MPPUS were also categorized by using the criteria by Chow et al. [43], which entail the establishment of four categories (Casual Users, Regular Users, At Risk Users, and Problematic Users), with the 15, 80, and 95 percentiles. In some analyses, we reduced these categories to two (Normal Users and Problem Users). In this sense, Kalhori et al. [36] establish that the maximum score that best discriminates between dependence and non-dependence would be a score of 160 among university students, considering two unique categories; Sahin et al. [18] and Foerster et al. [28] consider that cell phone use is a continuum in which a higher score is always associated with a greater problematic use without need for classification; Park and Lee [45] consider the 25% above and below as criteria of differentiation between addict users and non-addict users; and Smetaniuk[38] uses segments of “Low to Moderate” (27 to 76 points), “Moderate to High” (77 to 126 points), and “High to Severe” (above 127 points).

With these categories, we obtained a **prevalence of *20*.*5***% of Problem Users (the sum of At Risk Users and Problematic Users), with significant differences in age, primarily 16-25-year-olds and up to 35-year-olds, and in the higher level of parental education. This prevalence percentage comes close to those obtained in other studies using the MPPUS, such as the study by López-Fernández et al. [34], who, using the same criteria, obtain a total of 20.1% among Spanish adolescents and 20.5% among British students [35]; Kalhori et al. [36] obtain 23.4% of students with dependency; Park and Lee [45] indicate 13.9% of addiction in students; and Leung [20] and Leung [27] obtain 28.7% and 27.4% of students with addiction after adapting the MMUS (MPAI).

Overall, the logistic regression analysis shows us that having a higher level of education, spending a greater number of hours on the phone every day, being male, and mostly being aged up to 35 years and, to a lesser extent, up to 45 years can increase the probability of being a Problem User. The remaining factors, are variables that do not have predictive value but are related to problematic use. Actually, age and the hours of daily cell phone use are the variables with the greatest role and significance. Gender and level of education are shown to play a lesser role in our study.

In the case of gender, although the study showed that being male is a significant factor for classification as a Problem User, the fact that we were unable to find significant differences in prevalence throughout this study requires us to be cautious regarding this fact, which is not in line with other studies [20,31,33–35,37,45]. In other studies, problematic cell phone use or addiction always has greater proximity to women, being inclined to social anxiety in interpersonal relationships [46], in which their self-identity and need to belong is at play [47], leading some researchers to suggest that cell phones are a vehicle for addiction [4,6,44,48,49].

By contrast, age is capable of predicting problematic cell phone use, with high significance up to 35 years of age and, to a lesser extent, up to 45. Similarly, it maintains a progressively inverse relationship (r = -0.304, p = 0.000) with this problematic use. This finding is in line with results from other studies, such as those by Montag et al. [33] (r = -0.32, p = 0.02) and Smetaniuk[38], (r = -0.35, p<0.01), in which there is coincidence in that its use diminishes with increased age [18,24,34,35,50–56].

Regarding hours of use, its important predictive capability is not new and aligns with many other studies [20,24,31,57]. In our study, this relationship appears at all ages, though cell phone use diminishes with age, being higher at younger ages, as shown by other investigations [35,50,52–55]. However, recent studies indicate that it is not the total time but rather the type of use during this time that differentiates a user with addiction, whose use is unfocused and without specific goals, from a user who uses it sparingly, with a more specific task and application-focused utilization [58,59].

The parental level of education also has not been shown to be a predictor in our study, though, on the other hand, a higher level of education significantly stands out in prevalence. Nonetheless, studies are conflicting in this respect. Thus, Sánchez-Martínez and Otero [60], Billieux[4], Mazaheri and Najarkolaei[55], and Tavakolizadeh et al.[61] find a direct relationship between students of families with higher cultural and economic levels and dependency or problematic cell phone use. By contrast, Sahin et al. [18], López-Fernández et al. [34], and Leung [20] observe an inverse relationship between cell phone use and parents’ education and socioeconomic level. Thus, it is possible that we are faced with a double education pattern with different ways of use related to socioeconomic and cultural level.

The same can be said for drug use; although it does not have predictive capabilities, illegal drug use nonetheless significantly stands out among users aged 16 to 25 years, which is in line with what is shown by Sánchez-Martínez and Otero [60]. These authors find a significant relationship between students and cell phone abuse, being a smoker, and consuming cannabis and other drugs. This finding also aligns with what is shown by Toda et al. [29], who conclude that there is a relationship between cell phone abuse and smoking but who do not find such relationship with alcohol consumption. However, López-Fernández et al. [34] confirm such a relationship with nicotine and alcohol.

Finally, the number of friends with whom the user stays in touch, the quality of the data terminal, and the time of cell phone ownership are not shown to have predictive value in this study, though the first two are observed to be related to problematic use. In this respect, Billieux et al. [8] and Sahin et al. [18] find that a longer cell phone possession time progressively relates to problematic use, just as the number of friends is an indicator of the intensity and dependence of the social network [47,49,62].

Given the data of this study, we consider that problematic expressions of cell phone use would share criteria with recognized behavioral addictions such as gambling. This assumption is based on the observation of the presence of behavioral repertoires reported for gambling, uncluding: a) The quest for and progressive increase in behavior with the goal of achieving well-being or avoiding restlessness, b) endangering or losing personal and social relationships, work, or studies, c) repeated fruitless efforts to avoid the behavior, d) worry and persistent thoughts with a constant search for occasions and opportunities, e) the maintenance of the behavior despite the damage and prejudice that it provokes, e) denial of the dependency, and agitation and irritability when consumption or use is prevented, etc…. Similarly, we find parallels with some of the indicators of behavioural addiction such as loss of control, mood alterations, personal harm or conflicts in the environment, the tendency to relapse, dependency, tolerance with an increasing need for time and commitment, neglect of and interference with daily activities, automatism, the maintenance of the behaviour despite the negative effects observed by the environment, and abstinence reflected in irritability and discomfort [63–66].

In other words, a more in depth analysis is needed to confirm whether in problematic cell phone users we are facing a new type of addiction that would progressively affect large segments of the population shaped by different lifestyles and cultural levels that would give way to different problematic expressions. Based on our prevalence data, this potential addiction by listed degrees, depending on its severity, is as follows: At Risk Use, from which problems could also result, would affect 15.4% of the population, and Problematic Use, which would be the addiction proper, would have a prevalence of 5.1% of affected individuals. It is thus relevant that although there is an important incidence in the young population, we should not neglect the problems in the adult population.

Nonetheless, further research that would establish new causal relationships, that would consider variables that intervene, and that would stipulate the specific habits associated with this addiction of cell phones as a whole is still pending. Although the data in this study were obtained via a survey compared to recent data collection systems and knowing that self-perceptions tend to overestimate usage [23,33,58,59,67–69], we still cannot underestimate the self-awareness of excess and abuse that is already present in a problematic user.
